# Does Telerehabilitation Help in Reducing Disability among People with Musculoskeletal Conditions? A Preliminary Study

**DOI:** 10.3390/ijerph19010072

**Published:** 2021-12-22

**Authors:** Hana Alsobayel, Faris Alodaibi, Ali Albarrati, Norah Alsalamah, Fadwa Alhawas, Ahmed Alhowimel

**Affiliations:** 1 Department of Rehabilitation Sciences, College of Applied Medical Sciences, King Saud University, Riyadh 11433, Saudi Arabia; falodaibi@KSU.EDU.SA (F.A.); albarrati@KSU.EDU.SA (A.A.); 2 Research Chair for Healthcare Innovation, Department of Rehabilitation Sciences, King Saud University, Riyadh 11433, Saudi Arabia; 3 Saudi Physical Therapy Association, Riyadh 11433, Saudi Arabia; NM.alsalamh@gmail.com (N.A.); coachfadwa369@gmail.com (F.A.); 4 Department of Health and Rehabilitation Science, Sattam Bin Abdulaziz University, Al-Kharj 11942, Saudi Arabia; a.alhowimel@psau.edu.sa

**Keywords:** telerehabilitation, physiotherapy, musculoskeletal, Saudi Arabia

## Abstract

*Introduction*: During the coronavirus pandemic, an initiative was launched in Saudi Arabia to provide telerehabilitation for people with musculoskeletal conditions who were unable to access in-person physiotherapy due to the associated lockdown. The purpose of this study was to explore the therapeutic impact and acceptability of telerehabilitation among the Saudi population. *Methods*: Ninety-five participants were recruited through an online advertisement and received a physiotherapy consultation and interventions via an online video conference platform (Google Meet). Following screening for red flags, participants received tailored education and conditioning exercises 2–3 times a week for 6 weeks. Outcome measures were recorded at baseline and 6 weeks and comprised the Pain Self-Efficacy Questionnaire, Patient-Specific Functional Scale, Musculoskeletal Health Questionnaire and a satisfaction survey. *Results*: The most frequent musculoskeletal conditions reported were lower back (37%), knee (14%) and neck (10%) pain and post-operative conditions (15%). Participants showed significant improvements in outcomes at the end of the program (*p* < 0.001) with effect sizes ranging from 0.6 to −1.9 and reported a high level of satisfaction with the telerehabilitation intervention. *Conclusion:* This study showed that telerehabilitation was an acceptable method of providing physiotherapy interventions for patients with musculoskeletal conditions in Saudi Arabia.

## 1. Introduction

Musculoskeletal pain is considered a major burden for communities [1]. It is associated with marked impairment in functional activities and participation in work and other activities of daily living [2]. Physiotherapy interventions for patients with musculoskeletal conditions are designed to decrease pain, improve function, promote activity and self-management and address the physical limitations and beliefs and behaviors associated with musculoskeletal conditions. Most often, these physiotherapy interventions are provided in an outpatient setting on a one-on-one basis with the physiotherapist and patient.

Telerehabilitation denotes the use of technology in healthcare to enable “healing at a distance” [3]. It is a promising option to overcome the shift in demographics to an aging population and the increase in chronic diseases which are associated with high demand for rehabilitation services. It has the potential to reach rural and difficult environmental areas [3,4]. Despite its potential, the adoption of telerehabilitation into clinical practice has been slow. One reason for slow adoption rates is thought to be the lack of acceptance by patients and physiotherapists to engage in telerehabilitation [5,6].

During the recent outbreak of the COVID-19 pandemic, World Physiotherapy published resources for member organizations to support physiotherapy practice during and after the lockdown [7]. Telerehabilitation has provided a safe option for the continued delivery of treatment for many patients with conditions that would usually require in-person attendance in an outpatient setting.

Previous research has shown that, compared to in-person assessment, online physiotherapy assessment for musculoskeletal pain has good concurrent validity and excellent reliability [8,9] and, from the few studies available, it has achieved similar treatment outcomes for patients with musculoskeletal conditions [10,11,12,13]. Furthermore, both patients and therapists were found to be willing to adapt to the change in treatment delivery from in-person to online [4,14] and greater adherence to home exercise programs was seen for those receiving telerehabilitation [15].

In Saudi Arabia, telemedicine in general is in its infancy. However, the rapid growth and advancement of digital technology along with robust infrastructure in Saudi Arabia makes it possible to successfully apply telerehabilitation. The health authorities in Saudi Arabia realized the importance of telehealth long before the COVID-19 pandemic. The National Health Information Center published national guidelines for telehealth, which were updated in 2020 [16]. During the outbreak of COVID-19, access to in-person health consultations, including physiotherapy, for non-urgent conditions, was vastly reduced due to the lockdown instituted to reduce the spread of infection. In an attempt to address this issue, the Saudi Physical Therapy Association launched an initiative where they partnered with one of the private physiotherapy practices in Riyadh, Saudi Arabia, to provide telerehabilitation. The aim of this study was explore the therapeutic impact and acceptability of telerehabilitation physiotherapy for patients with musculoskeletal conditions in Saudi Arabia.

## 2. Materials and Methods

### 2.1. Study Design

A prospective quasi-experimental pre–post intervention repeated measure design was undertaken, from May 2020 until July 2020, to measure treatment outcomes and participant satisfaction for patients with musculoskeletal conditions receiving telerehabilitation. The study was approved by the institutional review board of Prince Sattam Bin Abdul-Aziz University (No: RHPT/020/022). All participants gave informed verbal consent during the first telerehabilitation session.

### 2.2. Participants and Setting

It was determined that a sample size of 122 patients was required. The sample size was calculated using G.Power 3.1 based on determinants of a *t*-test of a single group with effect size = 0.3, α = 0.05. The effect size was estimated by authors to be small based on Cohen’s d range, where 0.2 to 0.5 is considered a small effect size [17]. One hundred patients consented to participate in this study. The study involved a single private physiotherapy practice in Riyadh, Saudi Arabia. Patients referred to this practice were eligible for inclusion if they were 18 years of age or greater and presented with a non-urgent musculoskeletal condition that was deemed appropriate for non-surgical management. Patients were excluded if they presented with red flags (e.g., unexplained weight loss, autonomic signs, sudden change in signs and symptoms), cognitive, hearing or visual impairments that would preclude safe and adequate participation in online sessions or an inability to communicate adequately in Arabic. Patients were also required to have access to an internet-enabled computer or similar device. The physiotherapists providing the telerehabilitation interventions had to be licensed and have at least one year of experience in treating musculoskeletal conditions. The online physiotherapy program was advertised on social media, mainly via the official Twitter account of the Saudi Physical Therapy Association.

### 2.3. Intervention

The telerehabilitation intervention was provided using Google Meet and followed the Governing Rules of Telehealth (Telemedicine) in Saudi Arabia published by the Saudi Health Council in 2020 [16]. To ensure privacy, none of the sessions were recorded and participants’ data were de-identified and stored by one of the study investigators (AA). All physiotherapists providing the telerehabilitation signed a privacy agreement before they started the telerehabilitation interventions.

The first telerehabilitation session included collection of demographic and descriptive data and baseline outcome measures by the data management team, who were distinct from the treating physiotherapists. All participants were then assessed by their assigned physiotherapist, who had received a brief history about the participant before the session. In this and subsequent telerehabilitation sessions, participants received tailored education (e.g., information about their specific condition, how to manage pain and the effect of activity on pain and disability) and conditioning exercises (e.g., strengthening, flexibility, aerobic training). Each participant received telerehabilitation from their assigned physiotherapist 2–3 times a week for 6 weeks to monitor their response to interventions and for exercise progression. Session duration ranged from 20–40 min.

### 2.4. Outcome Measures

Outcome measures were collected at baseline and 6 weeks by the data management team to ensure privacy and accuracy. The Pain Self-Efficacy (PSE) questionnaire is a 10-item questionnaire developed to measure the confidence of people with ongoing pain have in performing their daily activities despite their pain, with acceptable validity and reliability [18,19]. Each item is rated on a 0–6 scale and used to generate a total score with a range of 0–60, with higher scores reflecting greater confidence. The Arabic version of PSE showed very good reliability (ICC = 0.79; Cronbach’s alpha (α) of 0.90) [19]. The Patient-Specific Functional Scale (PSFS), a valid, reliable and responsive measure, was used to assess participants’ activity limitation and functional ability [20,21]. This scale involves patients identifying up to 3 activities they are unable to perform or are having difficulty with and rating the level of difficulty associated with each activity on a 0–10 scale. A mean score, with a range of 0–10, is calculated by adding the individual item scores and dividing by the number of activities, with higher scores indicating higher functioning. The Arabic version of the PSFS had very good reliability (ICC = 0.86) [21]. The Musculoskeletal Health Questionnaire (MSK-HQ) is used to measure symptoms and quality of life and was specifically designed for the musculoskeletal population and has been shown to have good psychometric properties [22]. The MSK-HQ comprises 14 questions with a score of 0–4 possible for each item, and a total score range of 0–56, with higher scores reflecting better quality of life. The Arabic version of MSK-HQ showed very good reliability (ICC = 0.94; Cronbach’s alpha (α) of 0.88) [23].

Participant satisfaction was assessed at the end of the intervention period using a purpose-designed survey comprising six questions with responses given on a five-point Likert scale. These questions addressed participants’ satisfaction with the online sessions, the weekly follow up and their communication with the physiotherapist. The feedback from participating physiotherapists was also explored by asking 1 question about their satisfaction with the experience and was measured on 5-point Likert scale (1 = not satisfied to 5 = very satisfied). They were also asked about the possibility of using telerehabilitation in future and they answered yes, no or maybe. An open-ended question was also provided to allow them to write their feedback.

### 2.5. Analyses

Data were described descriptively by calculating means and standard deviations for continuous data and frequencies and percentages for categorical data. Paired-sample *t*-tests were used to compare the mean differences between baseline and follow-up scores for the three main outcome measures. Effect size was calculated by using Cohen’s d formula [23]. Analyses were conducted using the Statistical Package for the Social Sciences (SPSS, Version 25). Significance was set at *p* < 0.05. The data were normally distributed, therefore, parametric statistics were applied.

## 3. Results

A total of 100 patients were recruited to this study. Five were excluded since they did not consent to participate. The majority of participants were male (*n* = 83; 87%), the mean age was 33 years and 48 (51%) lived in Riyadh (Table 1). The most frequent musculoskeletal condition for which participants were seeking physiotherapy intervention was lower back pain (Table 1). Fourteen participants had post-operative conditions such as joint replacement surgery, ligament repair, spine fixation, osteotomy or arthroscopy.

**Figure 1:**
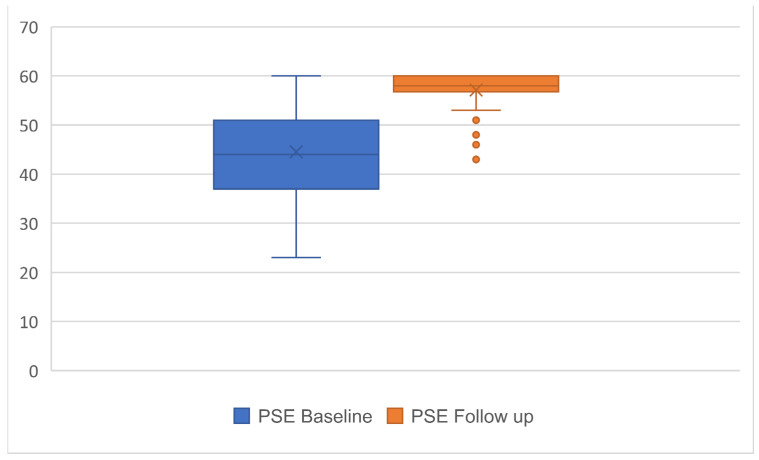
Boxplot of mean and SD for Pain Self-efficacy (PSE) scores at baseline and follow-up.

**Figure 2:**
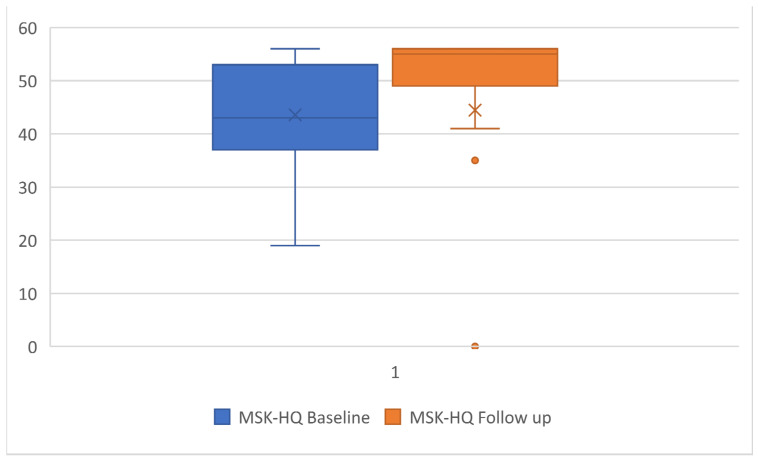
Boxplot of mean and SD for Musculoskeletal Health Questionnaire (MSK-HQ) scores at baseline and follow-up.

**Figure 3:**
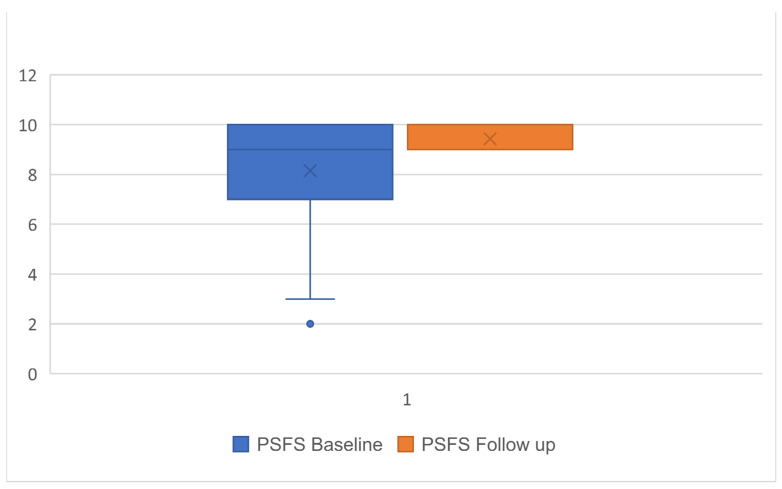
Boxplot of mean and SD for Patient-Specific Functional Scale (PSFS) scores at baseline and follow-up.

The mean (SD) time between baseline and follow-up was 39 ± 9 days, with 17 (18%) lost to follow up and 78 (82%) participants with completed data. Significant improvements from baseline to follow-up were observed for all three outcomes with a large effect size [17] ranging between 0.6 and −1.9 (*p* < 0.001) (Table 2; Figure 1, Figure 2 and Figure 3), with the highest effect size observed in the PSE scale.

**Figure 4:**
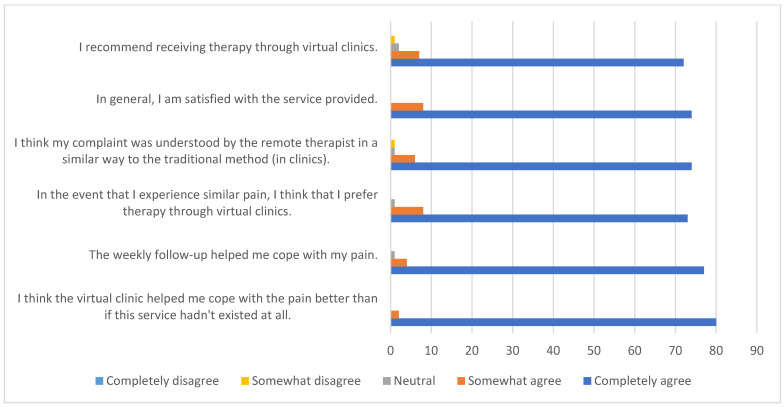
Satisfaction levels related to the telerehabilitation service.

Responses to the purpose-designed survey regarding participant satisfaction with the telerehabilitation service are summarized in Table 3 and Figure 4. Overall, participants’ responses reflected a high degree of satisfaction with telerehabilitation.

Participating therapists (*n* = 34), when asked about their feedback about their experience, were very satisfied (*n* = 20; 59%), satisfied (*n* = 11; 32%) or neutral (*n* = 3; 9%). When they were asked about conducting telerehabilitation in future, the majority answered “yes” (*n* = 31; 91%) or “maybe” *(n* = 3; 9%). The therapists also provided comments on their experience, and all of their comments were positive. They provided some concerns, including not being able to do physical examination, the short time of the session (i.e., 30 min) and being unsuitable for pediatrics. They also provided some recommendations for improvement, such as increasing the duration, including virtual reality and linking to an exercise program.

## 4. Discussion

This study examined the therapeutic impact and acceptability of physiotherapy assessment and interventions for patients with musculoskeletal conditions provided via telerehabilitation during the period of lockdown during the COVID-19 pandemic. Significant improvements were seen in pain, disability and health status as measured using the PSE questionnaire, PSFS and MSK-HQ from baseline to the end of the telerehabilitation intervention period, and a high degree of satisfaction with the telerehabilitation intervention was expressed by participants. These results support the positive impact on participants’ pain and function and acceptance of online physiotherapy for the management of patients with musculoskeletal conditions. Participants came from more than six districts out of the country’s 13 districts. This suggests that telerehabilitation provided easier access to physiotherapy care from wide geographical locations within the country, which supports the country’s healthcare reform to provide equitable and accessible healthcare services [24]. Easier access to healthcare may also improve adherence, which was suggested by Ku et al. [25], as they applied telerehabilitation in Hong Kong during the pandemic lockdown, and showed high adherence to the telerehabilitation (*n* = 1246), including physiotherapy and other rehabilitation services. The study also showed high levels of satisfaction from patients and rehabilitation professionals [25].

From the therapists’ perspective, this study showed that almost all therapists were satisfied with the experience, however, they highlighted some challenges, such as not being able to conduct hands-on assessment or treatment. It has been reported that online consultation and management may face challenges compared to conventional face-to-face encounters. Adapting assessment and management is required in order to compensate for the lack of a hands-on approach [26].

The results of this study, demonstrating the potential of physiotherapy provided via telerehabilitation for patients with musculoskeletal conditions, have implications for future clinical practice. Telerehabilitation may be a viable option for providing physiotherapy assessment and interventions for non-surgical patients suffering from musculoskeletal disorders who reside outside urban communities, where access to clinics can be difficult and time-consuming for both patients and clinicians [10,25]. Previous studies found home-based telerehabilitation may serve as an alternative option for delivering outpatient rehabilitation for patients suffering from chronic musculoskeletal pain (i.e., neck, shoulder, back) compared to the usual healthcare or home-based rehabilitation [11,12,27]. In a systematic review, Mani et al. found telerehabilitation by physiotherapists to be technically feasible, with “good to excellent” validity and reliability [8]. Furthermore, Cottrell et al. performed a systematic review and meta-analysis, analyzing the effectiveness of real-time telerehabilitation with MSK conditions when compared to standard face-to-face practice; this review demonstrated telerehabilitation to be effective in improving physical function, disability and pain [13]. With regard to clinical diagnostic accuracy, there are certain areas (e.g., range of movement) where telerehabilitation is superior to face-to-face consultation [28]. Increasing evidence suggests that telerehabilitation may be considered as a substitution for face-to-face interventions for reducing pain and improving physical function, daily life activities and quality of life in patients affected by MSK disorders [29]. However, one must be cautious when interpreting the evidence and examine its strength.

Another benefit of telerehabilitation has been shown by a group of researchers in Australia who have implemented a telerehabilitation service in an advanced physiotherapy clinic for screening programs and provided patients with a single facility point where they could access relevant healthcare services [10]. This has resulted in an increasing number of patients who visited this clinic for different telerehabilitation services. Other reports showed that telerehabilitation consultation for MSK pain has good to excellent reliability and validity [29].

Home-based telerehabilitation services remain relatively novel within Saudi Arabia, despite the excellent internet coverage for most cities in the kingdom. The ability to provide telerehabilitation services directly into the patients’ homes may open opportunities to reshape the traditional methods of providing healthcare for patients with musculoskeletal disorders without increasing the burden on the healthcare system and reducing healthcare and resource utilization [29,30,31,32]. Home-based telerehabilitation has been used for people with different chronic conditions and showed promising results, such as the pilot study by Chen et al. [33] where they delivered home-based exercises using virtual games that were monitored by therapists virtually. The study demonstrated that the efficacy of the therapist-monitored interactive telerehabilitation system was similar to that of conventional therapist-delivered face-to-face therapy to improve balance in those with chronic stroke [33]. Another example of home-based telerehabilitation for chronic conditions is the study by Hernando-Garijo et al. [34], a randomized controlled clinical trial investigating video-based aerobic exercise in women with fibromyalgia during the lockdown caused by the COVID-19 pandemic. The results showed that the intervention group achieved statistically significant improvements in pain intensity, mechanical pain sensitivity and psychological distress compared to a control group [34]. Telerehabilitation has also been used to support home-based exercises following hip surgery in the elderly, where exercises were delivered by caregivers at home instead of a health professional and guided by video recordings [35]. This controlled study showed that, regardless of the intervention received, both groups improved function, but the telerehabilitation program was superior at three months for improving function [35].

The advancement in technology and availability of fast internet connections and smart devices make it possible to expand the use of telerehabilitation for different conditions. Training and education are required in order to maximize the benefits of telerehabilitation as well as re-examining the regulations, policies and reimbursement models within the healthcare system [36]. More research is required to examine the efficacy of such an approach in the long term and for other conditions.

## 5. Study Limitations

The current study was quasi-experimental, and did not include a control group. This limits the generalizability of results. However, it is exploratory in nature and explored the acceptability and the therapeutic impact on the Saudi population. The study provided baseline information that could guide clinical trials in future. The study also relied on self-report from participants, making it difficult to validate the changes in their function. However, using several outcome measures with consistent results suggests clinically meaningful changes.

## 6. Conclusions

The growing evidence on the effectiveness of digital health, particularly telerehabilitation, is encouraging. It has potential in improving patients’ experience and accessibility to healthcare. The current study has shown that telerehabilitation has a positive therapeutic impact on pain and function and was acceptable method of delivering physiotherapy services for patients who presented with musculoskeletal conditions. The advanced digital infrastructure in Saudi Arabia made it possible to adopt this approach more widely in physiotherapy practice. More research is needed to examine the long-term impact of telerehabilitation. In addition, policy makers and regulatory bodies need to re-examine education, training and reimbursement of services to maximize the benefits of telerehabilitation.

## Figures and Tables

**Table 1:** Participants’ demographic and descriptive data (*n* = 95).

Characteristics		*n* (%)
Age, years, mean (SD)		33 (8)
Gender, male		83 (87)
Marital status	Single	53 (56)
	Married	42 (44)
Level of education	Illiterate	1 (1)
	High school	3 (3)
	College	91 (96)
Employment status	Employed	81 (85)
	Unemployed	12 (13)
	Retired	2 (2)
Region	Riyadh	48 (51)
	Qassim	9 (10)
	Tabuk	6 (6)
	Jazan	6 (6)
	Eastern region	4 (4)
	Jeddah	4 (4)
	Other	18 (19)
Smoker		28 (28)
Body mass index, weight (kg)/height (m)^2^, mean (SD)		25 (4)
Past medical history	Hypertension	7 (7)
	Diabetes	6 (6)
Musculoskeletal disorder	Lower back pain	35 (37)
	Knee pain	13 (14)
	Neck pain	9 (10)
	Shoulder pain	7 (7)
	Ankle pain	5 (5)
	Hip pain	2 (2)
	Elbow pain	2 (2)
	Post-operative condition	14 (15)

**Table 2:** Baseline and follow-up outcome measurements.

Outcome Measures	Baseline Mean (SD)	Follow Up Mean (SD)	Effect Size (Cohen’s d)
Pain Self-Efficacy Questionnaire	44.6 (9.6)	57.1 (3.6)	−1.9 *
Patient-Specific Functional Scale	8.1 (2.1)	9.4 (0.5)	0.6 *
Musculoskeletal Health Questionnaire	43.6 (9.3)	54.2 (3.6)	−1.4 *

* *p* < 0.001.

**Table 3:** Satisfaction levels related to the telerehabilitation service.

Statements	Completely Agree *n* (%)	Somewhat Agree *n* (%)	Neutral *n* (%)	Somewhat Disagree *n* (%)	Completely Disagree *n* (%)
I think the virtual clinic helped me cope with the pain better than if this service hadn’t existed at all.	76 (80)	2 (2)	0 (0)	0 (0)	0 (0)
The weekly follow-up helped me cope with my pain.	73 (77)	4 (4)	1 (1)	0 (0)	0 (0)
In the event that I experience similar pain, I think that I prefer therapy through virtual clinics.	69 (73)	8 (8)	1 (1)	0 (0)	0 (0)
I think my complaint was understood by the remote therapist in a similar way to the traditional method (in clinics).	70 (74)	6 (6)	1 (1)	1 (1)	0 (0)
In general, I am satisfied with the service provided.	70 (74)	8 (8)	0 (0)	0 (0)	0 (0)
I recommend receiving therapy through virtual clinics.	68 (72)	7 (7)	2 (2)	1 (1)	0 (0)

## Data Availability

Data were de-identified and stored by one of the study investigators (AA).
